# Emotional Processing, Coping, and Cancer-Related Sickness Symptoms in Breast Cancer Survivors: Cross- Sectional Secondary Analysis of the REPAT Study

**DOI:** 10.21203/rs.3.rs-3164706/v1

**Published:** 2023-07-19

**Authors:** Keren Harel, Johanna Czamanski-Cohen, Miri Cohen, Karen L. Weihs

**Affiliations:** University of Haifa; University of Haifa; University of Haifa; University of Arizona

**Keywords:** breast cancer survivor, cancer-related sickness symptom, approach coping, acceptance, avoidance coping

## Abstract

**Purpose:**

The study aims to provide a better understanding of the relationship between emotional processing, coping, and cancer-related sickness symptoms.

**Methods:**

The study used a cross-sectional, secondary analysis of data from 179 Israeli Jewish women who were breast cancer survivors (BCS) 3 to 18 months after completing primary treatment and who participated in a larger randomized controlled trial (REPAT study). Data were collected at baseline. Participants completed questionnaires measuring emotion acceptance, situational approach, avoidance coping, and cancer-related sickness symptoms (depression, fatigue, and pain) and a performance measure of emotional awareness. Hierarchical linear regressions were performed, controlling for background variables.

**Results:**

Participants experienced significant clinical depression (51.7%), cancer-related fatigue (CRF, 78.8%), pain interference (78%), and pain intensity (66%) levels. There were strong correlations between cancer-related symptoms. After controlling for confounders, emotional processing (acceptance) was negatively associated with depression, and avoidance coping was positively associated with depression, CRF, and pain interference (i.e., higher use of avoidance related to higher cancer-related symptoms; higher acceptance was associated with lower depression). Emotional awareness and coping by approaching emotions were not related to cancer-related symptoms.

**Conclusions:**

The BCS posttreatment period presents the challenge of dealing with elevated cancer-related symptoms. Regardless, BCS who used high emotional processing levels—especially acceptance of emotion and lower reliance on avoidance to cope—experienced fewer cancer-related symptoms.

**Implications for Cancer Survivors:**

Professionals should recognize the potential role of emotional processing and avoidant coping relative to cancer-related symptoms and recognize their patterns in posttreatment patients.

## Introduction

Worldwide survival rates for breast cancer are increasing. The 5-year survival rates were 85 to 87% between 2010 and 2014 in Western European countries [1] and, in the United States, close to 90% in 2011, with Stage 1 5-year survival approaching 100% [2]. It was 89.2% and 84% in Israel between 2008 and 2014, for invasive breast cancer in Jewish and Arab women, respectively [3]. However, in the posttreatment period, survivorship poses several challenges, including cancer-related sickness symptoms, such as depression, fatigue, and pain associated with lower quality of life (QoL) [4].

Cancer-related sickness symptoms may co-occur in breast cancer survivors (BCS) [4], substantially affecting many areas of life. The prevalence of depressive symptoms varies greatly [5]. Based on the results of 17 studies in a systematic review involving BCS 1-year postdiagnosis, the depressive symptoms prevalence ranged from 9.4–66.1%, with 22% reporting severe symptoms [5]. Despite inconsistent findings, a few meta-analyses have associated depression with higher rates of cancer recurrence, all-cause mortality, and cancer-specific mortality (e.g., [6]), emphasizing the importance of treating depression among BCS. Cancer-related fatigue (CRF) may affect the physical, cognitive, and affective domains, limiting survivors’ QoL and ability to adjust to postcancer life, including the ability to return to work [7]. According to a meta-analysis [7], approximately one in four BCS suffer severe fatigue (26.9%); some cases last 5 to 10 years and negatively affecting QoL. Survivors report varying degrees of pain that may interfere with their daily activities and persist following treatment. Another meta-analysis [8] showed that 29.8% of patients who underwent surgery (*n* = 3,746), 27.3% who completed radiation therapy (*n* = 15,019), and 21.8% who were subject to various combinations of breast cancer treatments (*n* = 135,437) reported pain.

Depression, CRF, and pain are intercorrelated [4] and associated with several sociodemographic variables, including lower income [9] and education levels [10], not having a partner [7], being younger [11], comorbid diseases, and long, rigorous treatment courses, including treatment combinations [11] with chemotherapy [7]. A systematic review demonstrated that coping and emotional regulation directly affect psychological and physiological symptoms (e.g., depression, CRF, and anxiety) [12]. Despite this relationship, pain symptoms were not included in the review [12], and fatigue was reported in only two studies [13, 14]. However, emotional processing received limited research attention.

*Emotional processing* involves several overlapping processes that may (or not) be conscious. It is an active attempt to acknowledge, explore meanings, validate, reflect, and verbally (or not) label, reappraise, be aware of arising emotions and somatic sensations, and understanding one’s and others’ emotional experiences [15, 16]. In this process, a person must nurture feelings and accept that emotions provide important information. Emotions might change their understanding and decrease their guilt, shame, or fear, leading to a more comprehensive understanding or reducing emotional disturbances and arousal. By engaging with differentiated and processed emotional experiences and adopting an accepting approach, they might better express and cope with emotions [17]. Pascual-Leone and Greenberg [17] argued that accepting emotions and exhibiting an agency state of performance (e.g., “I can cope”) are crucial to successful emotional processing. In poor emotional processing, avoiding or repressing emotions for prolonged periods may result in the use of avoidant coping styles [18].

Approach and avoidance coping are different motivational systems that influence behavior in response to stressors and challenging events. *Approach coping* involves cognitive or behavioral efforts to engage actively with the stressor or its emotional distress. It involves accepting, managing, and confronting the stressor directly (e.g., seeking social support, solving problems, and reevaluating the situation). *Avoidance coping* is the tendency to distance oneself emotionally, cognitively, and behaviorally from stressors. An avoidance coping strategy may include withdrawal, distraction, denial, or suppression of stress-related thoughts and feelings [18].

Emotional processing has positive associations with health outcomes and QoL among BCS [19, 20]. Accepting emotions was found to predict more positive adjustment over time [19] and decreased emotional distress [20], nausea, and CRF [21]. Acceptance of emotions and emotional expression were associated with lower depressive symptoms [22]. Further, 95% of 43 studies (39 on breast cancer) in a systematic review measuring coping among mostly early-stage BCS showed coping patterns were independent predictors of depression, anxiety, and health-related QoL [23]. Another meta-analysis [24] associated engagement forms of coping, including acceptance and positive reappraisal, with more positive affect and higher psychological and physical health levels. Disengagement coping forms had higher negative affect, lower positive affect, and lower physical health [24].

These studies highlighted the positive impact of emotional processing, including accepting emotions and engaging coping strategies on QoL. However, studies showed mixed results. Some showed that coping strategies had no significant effect on BCS symptoms compared to controls [25]; others negatively associated active coping (5–15 years from breast cancer diagnosis) with mental QoL and did not find that positive coping predicted mental QoL [26]. Although engaging coping strategies may positively affect cancer-related symptoms and QoL, they also might be exhausting in a continual health crisis [26].

There is limited knowledge regarding the relationship between emotional processing, coping, and cancer-related sickness symptoms among BCS posttreatment. This study aimed to examine the relationship between emotional processing (emotional awareness and acceptance of emotions), coping (approach and avoidance coping), and cancer-related sickness symptoms (depression, CRF, and pain).

## Methods

### Participants and Procedure

This descriptive, cross-sectional study was conducted as part of a secondary analysis of the baseline data of the randomized controlled trial, “The Role of Emotional Processing in Art Therapy for Breast Cancer Patients (REPAT)” [27]. The REPAT study examined the role of emotional processing and the cholinergic anti-inflammatory pathway in reducing depression pain and CRF among BCS through art therapy intervention. Participants were recruited between May 2019 and March 2022. The study was conducted at the Integrative Oncology Medicine unit of the Davidoff Center for cancer treatment at Rabin Medical Center, Israel, as well as with the community of Israel. The study was approved by the Helsinki Committee of the Beilinson Hospital (0778-17-RMC) and the Ethics Committee of the Faculty of Welfare and Health Sciences at the University of Haifa (234/18).

The eligibility criteria were (1) adult female (> 18 years) BCS with primary, first-recurrence breast cancer (including recurrence of breast cancer) or second primary diagnosis of breast cancer; )2) any additional or replacement standard medical treatment for cancer (i.e., surgery, chemotherapy, radiotherapy, neo-adjuvant chemotherapy, endocrine therapy); (3) not less than 3 months after finishing chemotherapy or radiotherapy, not less than 1 month after surgery (lumpectomy, mastectomy, or reconstructive surgery), and no more than 19 months after the end of treatment; (4) able to complete assessments in Hebrew. Ineligibility criteria were (1) diagnosis or lifetime history of bipolar disorder, schizophrenia, or schizoaffective disorder; (2) fibromyalgia or chronic fatigue syndrome; (3) active suicidal plan; (4) dementia/other disorder that would preclude informed consent.

The medical staff of Davidoff Center recruited participants through phone calls and personal applications during follow-up appointments and collected informed consent and medical information. The REPAT team recruited through nonprofit organizations, social communities, social media, and word of mouth and collected medical information and Self-report questionnaires in the community. An equivalent of US$200 in new Israeli shekels was provided to each participant as compensation for their time attending all 11 REPAT sessions.

Two hundred eighty-seven BCS met the inclusion criteria and signed informed consent. Of them, 18 dropped out for various reasons (e.g., loss of follow-up, no interest, work, lack of time, and medical conditions). Another 28 participants were excluded due to blank questionnaires and logistic issues. In total, 241 participants were included in the REPAT study’s final baseline, of which a subsample of 179 Jewish participants was included in this analysis. A priori power analysis using G*Power for multiple regression analysis, given power = .80, α = .05 (medium effect size), and 12 variables (study and background) computed a total sample size of 127 participants [28].

Measures of emotional awareness; acceptance, approach, and avoidance coping; and pain interference and intensity were translated for the present study from English into Hebrew by two bilingual experts using the back translation method and tested in a pretest phase.

### Measures

#### Demographic and Medical Information

Sociodemographic (e.g., age, marital status, education, household income, religiosity), lifestyle habits (e.g., smoking, diet, alcohol consumption), and medical information (e.g., cancer diagnosis, cancer stage, cancer therapy, date of last treatment) were collected through participants’ self-reports or medical records review.

#### Emotional Processing and Coping

Emotional awareness was assessed using the Levels of Emotional Awareness Scale (LEAS) performance measure in its short (10-item) version [15]. In response to short, evocative interpersonal scenarios, participants write about their and the others’ emotional reactions, beginning with “I would feel. . ., and the other would feel. . .” Based on the degree of response to a specific emotion, the number of emotions described, and the total score of self and others, their responses are scored on a 0-to-5 scale. Higher scores reflect greater differentiation and awareness of emotional complexity in self and others. This study’s LEAS total score internal consistency was Cronbach’s alpha = .70.

The 13-item Acceptance of Emotions Scale was designed to measure the extent to which participants accept and nurture their feelings [20], for example, “I naturally and easily attend to my feelings.” Responses on a 10-point Likert scale assess how much each statement describes the participant’s feelings, from 0 (*not at all like me*) to 100 (*exactly like me*). This study’s internal consistency was Cronbach’s alpha = .94.

The Situational Approach and Avoidance Oriented coping scales [18] were adjusted to the experience of women coping with breast cancer. This 24-item situational approach scale consists of three subscales from the Coping Orientation to Problems Experienced (COPE) [29] and two from the Coping through Emotional Approach [30] scale. Situational avoidance coping is a 12-item scale with three subscales from the COPE scale [29]. An example statement from approach coping is, “I think hard about what steps to take,” and from avoidance coping is, “I turn to work or other substitute activities to take my mind off things.” Participants respond on a Likert scale from 1 (*I don’t do this at all*) to 4 (*I do this a lot*).

As Carver et al. [29] suggested, we performed an exploratory factor analysis of principal component analysis using all these scales to determine which factors capture most features and a Kaiser-Meyer-Olkin and Bartlett’s test of sphericity to check their sufficiency for factor analysis. Additionally, we conducted a scree plot presenting an eigenvalue of two factors. Accordingly, we examined a rotation method of varimax with Kaiser normalization (item loadings on the indexes) using two factors. Three items from the situational approach coping had low loadings and were dropped from further analysis. Items from the situational avoidance coping scale had good loadings and matched the original scale [18]. Further details of the factor analysis can be found in Supplementary Table 2. In this study, the internal consistency (after factor analysis) of the situational approach and avoidance coping scales, respectively, was Cronbach’s alpha = .91 and .72.

#### Cancer-Related Sickness Symptoms

Depressive symptoms were measured using the Center for Epidemiologic Studies-Depression (CES-D) scale (10 items), Hebrew translation and validation [31], using a frequency scale of 0 (*never*) to 3 (*most of the time*). Depression was assessed by referring to the extent the participant reported experiencing feelings such as “sad and depressed” in the last week. The suggested cutoff for clinical depression is 10 points [32]. This study’s internal consistency was Cronbach’s alpha = .79.

Cancer-related fatigue was measured using the Fatigue Symptom Inventory scale (13 items and one qualitative question), Hebrew translation [33], designed for measuring CRF intensity (four items), interference (seven items), and duration (two items) with QoL [34]. Example statements include “Rate your fatigue level on the day you felt most fatigued” and “Rate how much, in the past week, fatigue interfered with your general level of activity.” Intensity and interference are rated from 0 (*not at all fatigued/no interference*) to 10 (*extremely fatigued/extreme interference*). Duration is measured on a scale from 0 (*no days*) to 7 (*all days*) over the past week and by the percentage of time fatigue was present each day. The suggested cutoff to consider a patient clinically fatigued is three points [35]. The internal consistency in this study was Cronbach’s alpha = .95.

Pain was measured using the Patient-Reported Outcomes Measurement Information System (PROMIS). The six-item Pain Interference Scale (PIQ-6a) measured how much pain interfered with different aspects of life in the past 7 days, and the three-item Pain Intensity Scale 3a measured the pain’s intensity [36]. The pain interference Likert scale ranged from 0 (*not at all interfered*) to 4 (*very much interfered*), similar to the pain intensity Likert scale from 0 (*not at all intense*) to 4 (*extremely intense*). Example items included “To what extent did the pain interfere with your daily activities?” and “What was the intensity of your worst pain?” According to the PROMIS coding instructions, item-level calibration *t* scores are calculated for each measurement using REDCap auto-score based on raw scores [37, 38]. The cutoff for clinic pain interference and intensity is a *t* score above 50 [38]. This study’s internal consistency for pain interference and intensity, respectively, was Cronbach’s alpha = .95 and .96.

#### Data Analysis

We used IBM SPSS (version 25) to examine the study hypotheses. For all variables, descriptive statistics were first calculated using means and standard deviations. *T* tests and analysis of variance (ANOVA) were used to compare differences between study variables (depression, CRF, pain intensity, pain interference, emotional awareness, emotional acceptance, approach, and avoidance coping) and background variables (e.g., age, income, education, religiosity, marital status, children, time from end of treatment, and body mass index). Pearson correlations were used to assess correlations between the study variables. We conducted hierarchical linear regressions controlling for background variables to assess the adjusted variance of each cancer-related symptom (depression, CRF, and pain) explained by emotional processing variables (emotional awareness and acceptance) and coping strategies (approach and avoidance). We imputed an item mean within the scale for each participant with 20% or fewer missing responses in the scale [39].

## Results

Table 1 presents the participants’ demographics and medical details. Most were between 46 and 70 years old, married or in a long-term relationship, and had education levels of certificate (14 years of education) and above. About half were employed, and most earned average or above incomes. Most participants had been diagnosed with early-stage breast cancer and underwent surgery; about half received chemotherapy with radiotherapy. The average score of all participants was above the cutoff for clinical depression, pain, and CRF. Among the participants, 52% were clinically depressed, 78.8% were clinically fatigued, 78% showed clinical levels of pain interference, and 66% showed clinical levels of pain intensity. Acceptance of emotions was negatively related, and avoidance coping was positively related, to depression and CRF. This means that higher acceptance of emotions and lower avoidance coping were associated with lower depression and CRF. In addition, avoidance coping was positively associated with pain interference, meaning that lower avoidance coping was associated with lower pain interference. Emotional awareness and approach coping were not associated with cancer-related symptoms but with acceptance of emotions. Higher acceptance was associated with more approach coping and higher emotional awareness. Cancer-related sickness symptoms were positively and strongly correlated with each other. Table 2 presents the Pearson zero-order correlations and the study variables’ means.

The hierarchical multiple linear regression analysis is presented in Table 3. Study variables showed significant differences in *t* tests and ANOVA by income level and religiosity. Hence, they were controlled for in the regression analyses. Income and religiosity levels were entered in Step 1. Income level was negatively associated with depression and CRF, meaning that when income was lower, depression and CRF were higher. In Step 2, the independent variables of emotional awareness and acceptance and approach and avoidant coping were entered. Acceptance of emotions was negatively related to depression but not to CRF and pain (interference and intensity), meaning that the greater the acceptance of emotions, the lower the depression. Coping through avoidance was positively related to depression, CRF, and pain interference but not pain intensity. This means that the more coping through avoidance was used, the higher the depression, CRF, and pain interference were. Income level, acceptance of emotions, and coping through avoidance significantly explained 24.7% of the variance in depression, and income level and coping through avoidance significantly explained 21.2% of the variance in CRF. Additionally, coping through avoidance significantly explained 3.4% of the variance in pain interference, but the model was not significant.

## Discussion

Our findings indicate levels of depression, pain, and CRF above clinical cutoff points. Previous studies [5, 40] support these findings. Depression levels are within the upper range of findings from two meta-analyses [5, 41], and CRF levels are similar to previous results among BCS in Israel [42]. The CRF rates are relatively high compared to one meta-analysis [7], but that meta-analysis reported high heterogeneity. Compared to another meta-analysis [8], the present study’s pain levels are relatively high but similar for BCS in Israel [40]. However, as reported in the meta-analyses [5, 7], the prevalence of BCS cancer-related symptoms varied among studies.

Decreased medical monitoring, hyperalertness for signs of recurrence, and expectations that life will return to normal after cancer treatment [43] can contribute to high symptom levels. Other psychological factors, such as elevated anxiety levels [4], coping strategies, and pain catastrophizing [44], may influence these high levels. In line with previous studies [4], this study found strong intercorrelations among cancer-related symptoms (depression, CRF, and pain), generating a snowball effect in which bidirectional effects of symptoms on each other could amplify or increase the persistence of a given symptom. The interrelationships emphasize the importance of the present study and its main findings of lower depression related to high emotional acceptance—and higher depression, CRF, and pain interference related to high avoidance coping—as potential means of preventing that symptom snowball effect.

We found that coping through avoidance was associated with higher depression, fatigue, and pain interference. These findings align with previous studies that associated suppressing or avoiding emotions with higher negative affect and lower physical health (including increased CRF in a few studies [24]). Even fewer studies examined the associations between avoidance coping and pain; and found no associations [24]. Our study is the first to find an association between avoidance coping and pain interference. The fear-avoidance model [44] may explain this association.

According to this model, fear of pain (including pain anticipation) and pain catastrophizing may provoke the behavioral response of avoiding the threatening situation, leading to greater fear of pain and disability [44]. Physical disability (inactivity) affects the musculoskeletal, cardiovascular, and inflammatory-immune systems associated with increased pain [10], restoring a vicious cycle that may exacerbate the pain. A period of avoiding emotions can be an exhausting affair, especially during a continual health crisis, which requires considerable energy to suppress, avoid, and deny emotions [26]. As a result, coping through avoidance may be related to higher CRF. However, avoidance coping may be a useful short-term strategy (e.g., during acute treatment) to enhance a person’s perception of control and reduce emotional arousal [20]. An urge to control and manage emotional situations results from feelings of general anxiety strongly associated with CRF [4]. However, as this study’s findings indicate, excessive avoidance coping is associated with increased cancer-related symptoms during the posttreatment period.

The association between increased emotional acceptance and decreased depression in this study accords with previous findings. A meta-analysis showed that acceptance of emotions is related to lower levels of negative affect and depressive symptoms and higher well-being and health, particularly in studies of women with mixed stages of breast cancer [24]. Similar to the present study, no associations were found between acceptance and physical symptoms such as CRF and pain [24]. However, results were inconsistent; previous studies [21] found associations between accepting emotions and CRF across time points. Further, in a systematic review of psychological interventions aimed at reducing CRF, most studies demonstrated significant results from the interventions in reducing CRF. Despite these indications, there were significant differences among the studies, and definitive conclusions have yet to be reached [45].

Our study found no significant association between approach coping, emotional awareness, and cancer-related sickness symptoms. Nevertheless, a few longitudinal studies found an association between approach coping styles at one time and depression symptoms at other times (e.g., [22]). Despite the limited research, negative associations have also been found between active coping and quality of life (e.g., [26]). However, studies on emotional awareness and approach coping associated with CRF and pain are lacking.

Different assessment methods and diverse coping factors in other studies make it difficult to compare them with this study’s findings. In the present study, participants with higher emotional acceptance levels were more likely to use approach coping strategies. For example, participants high in emotional acceptance might express their emotions and thoughts to their loved ones (coping through approaching social support). According to Lazarus and Folkman [46], coping decreases during recovery from work stress or once a stressor has been resolved. Postprimary treatment may mark such a period when less approach coping is needed. Over the time since diagnosis, approach coping may be less widely used, but acceptance may play a major role. Sufficient emotional processing might lead to accepting one’s emotions [17]. Because emotional processing and coping vary during different breast cancer phases [24], they may require different emotional processing and coping strategies at different times.

## Limitations

The cross-sectional design of this study limited us from concluding directionality and causality. Further, the use of self-report measures targeting emotional processing and coping prevented us from fully portraying the participants’ experiences, expectations, and behaviors. Part of the data was collected during the COVID-19 pandemic, under mobility and contact restrictions and fear of being infected or infecting others. This may limit our findings’ applicability to breast cancer patients who do not have these restrictions. However, an Israeli study during COVID-19 among cancer patients found their mean psychological distress level to be rather low, and the patients were determined to continue treatment [47]. In addition, the results of the present study are limited to Israeli Jewish women BCS and may not demonstrate the same results in other populations.

## Conclusions and Implications for Cancer Survivors

This study’s findings of high clinical symptom levels and of intercorrelations between depression, CRF, and pain emphasize the need to understand emotional processing and coping and how they relate to cancer symptoms. These results confirm the findings of a meta-analysis [24] that associated avoidance coping with higher depression. However, we add novel findings regarding CRF and pain interference. In addition, these results support findings [22] of lower depression associated with acceptance of emotions. They may have practical implications: They add knowledge toward identifying BCS who may be at risk with low acceptance of emotions, excessive use of avoidance, and especially those from low income, affecting their rehabilitation, recurrence, and QoL [12]. This study also may contribute to oncology patients, helping them understand how accepting their emotions may regulate depression and improve mental and physical health and QoL. Future studies may consider examining interventions targeting emotional awareness, acceptance, and approach coping strategies.

## Figures and Tables

**Table 1:** Demographic and Characteristics of Jewish Breast Cancer Survivor Participants

	*n*(%)	*M*(*SD*)
Age (years)		
26–35	6 (3.4)	
36–45	35 (19.6)	
45–70	121 (67.6)	
Older than 70	17 (9.5)	
Education		
High school and below	33 (18.4)	
Certificate (14 years) and above	145 (81)	
Employed	103 (57.5)	
Income		
Below average	24 (13.4)	
Average	89 (49.7)	
Above average	64 (35.8)	
Married or in long-term relationship	127 (70.9)	
Have children	159 (88.8)	
Number of children		2.50 (1.36)
Level of religiosity		
Secular	99 (55.3)	
Traditional	49 (27.4)	
Religious	31 (17.3)	
Cancer stage		
Stage 0	5 (2.8)	
Stage 1	58 (32.4)	
Stage 2	72 (40.2)	
Stage 3	30 (16.8)	
Stage 4	8 (4.5)	
Treatment (combinations are possible)		
Chemo	111 (62)	
Radio	147 (82.1)	
Surgery	165 (92.2)	
Hormonal (Aromatase inhibitors, Tamoxifen, Letrozole)	133 (74.3)	
Biological	37 (20.7)	
Months from the end of treatment to the baseline		6.11 (3.97)
Months from diagnosis to the baseline		13.75 (5.7)
Body mass index		26.72 (5.27)
Smoke cigarettes	14 (7.9)	

**Table 2:** Pearson Zero-Order Correlation Between Study Variables

Variable	1	2	3	4	5	6	7	*M*(*SD*)
Depression								10.54 (5.86)
CRF Total^a^	.495**							57.69 (28.8)
Pain intensity	.248**	.403**						55.86 (12.68)
Pain interference	.300**	.494**	.789**					57.46 (9.83)
Avoidance coping^b^	.263**	.304**	.025	.201**				1.84 (0.47)
Approach coping^c^	−.040	.042	.008	.036	.102			3.20 (0.57)
Acceptance of emotions	−.366**	−.174*	−.114	−.123	−.047	.494**		63.56 (23.31)
Emotional awareness	.055	.116	−.084	−.079	.069	.089	.175*	28.50 (6.41)

Note

a CRF intensity mean = 5.00; *SD*= 2.29

b *N* = 165

c *N* = 146.

* *p* < .05

** *p* < .01 (2-tailed).

**Table 3:** Multiple Linear Regression Analyses of the Associations (Standardized Coefficients Beta) Between the Study’s Variables and Cancer-Related Symptoms

Model		Depression	CRF	Pain interference	Pain intensity
		β	β	β	β
1	Income level	−0.25**	−0.29**	−0.08	−0.12
	Religiosity	0.01	0.15	0.06	0.01
	*F*	4.55*	9.35**	0.80	1.07
	*R* ^2^	0.63	0.11	−0.00	0.00
2	Income level	−0.23**	−0.27**	−0.09	−0.14
	Religiosity	−0.01	0.10	0.03	0.01
	Avoidance coping	0.29**	0.30**	0.18*	−0.01
	Approach coping	0.13	0.03	0.09	0.06
	Acceptance of emotions	−0.44**	−0.12	−0.16	−0.11
	Emotional awareness	0.04	0.11	−0.13	−0.11
	*F*	8.47**	7.09**	1.81	0.76
	*R* ^2^	0.28	0.21	0.03	−0.01

*Note.* β = beta coefficient.

* *p* < .05

** *p* < .01.
